# Evolving Identification of Blood Cells Associated with Clinically Isolated Syndrome: Importance of Time since Clinical Presentation and Diagnostic MRI

**DOI:** 10.3390/ijms18061277

**Published:** 2017-06-15

**Authors:** Stephanie Trend, Anderson P. Jones, Sian Geldenhuys, Scott N. Byrne, Marzena J. Fabis-Pedrini, David Nolan, David R. Booth, William M. Carroll, Robyn M. Lucas, Allan G. Kermode, Prue H. Hart

**Affiliations:** 1 Telethon Kids Institute, University of Western Australia, Perth, WA 6008, Australia; stephanie.trend@telethonkids.org.au (S.T.); anderson.jones@telethonkids.org.au (A.P.J.); sian.geldenhuys@telethonkids.org.au (S.G.); 2 Cellular Photoimmunology Group, Infectious Diseases & Immunology, Charles Perkins Centre, University of Sydney, Sydney, NSW 2006, Australia; scott.byrne@sydney.edu.au; 3 Centre for Immunology and Allergy Research, Westmead Institute for Medical Research, University of Sydney, Sydney, NSW 2006, Australia; david.booth@sydney.edu.au; 4 Centre for Neuromuscular and Neurological Disorders, Perron Institute for Neurological and Translational Science, University of Western Australia, Sir Charles Gairdner Hospital, Perth, WA 6009, Australia; marzena.pedrini@perron.uwa.edu.au (M.J.F.-P.); robyn.lucas@anu.edu.au (W.M.C.); kermode@me.com (A.G.K.); 5 Immunology Department, Royal Perth Hospital, Perth, WA 6000, Australia; d.nolan@iiid.murdoch.edu.au; 6 Institute for Immunology and Infectious Disease, Murdoch University, Perth, WA 6150, Australia; 7 National Centre for Epidemiology & Population Health, Research School of Population Health, Australian National University, Canberra, ACT 0200, Australia; robyn.lucas@anu.edu.au

**Keywords:** multiple sclerosis, clinically isolated syndrome, immunology, pathology, B cells, NK cells

## Abstract

It is not clear how the profile of immune cells in peripheral blood differs between patients with clinically isolated syndrome (CIS) and healthy controls (HC). This study aimed to identify a CIS peripheral blood signature that may provide clues for potential immunomodulatory approaches early in disease. Peripheral blood mononuclear cells (PBMCs) were collected from 18 people with CIS, 19 HC and 13 individuals with other demyelinating conditions (ODC) including multiple sclerosis (MS). Individuals with CIS separated into two groups, namely those with early (≤14 days post-diagnostic magnetic resonance imaging (MRI); *n* = 6) and late (≥27 days; *n* = 12) blood sampling. Transitional B cells were increased in the blood of CIS patients independently of when blood was taken. However, there were two time-dependent effects found in the late CIS group relative to HC, including decreased CD56bright NK cells, which correlated significantly with time since MRI, and increased CD141+ myeloid dendritic cell (mDC2) frequencies. Higher CD1c+ B cells and lower non-classical monocyte frequencies were characteristic of more recent demyelinating disease activity (ODC and early CIS). Analysing cell populations by time since symptoms (subjective) and diagnostic MRI (objective) may contribute to understanding CIS.

## 1. Introduction

Clinically isolated syndrome (CIS) is often the first indication of multiple sclerosis (MS), though prediction of disease course in individuals with CIS is challenging, with few markers to predict conversion to MS [1]. When progression to MS does occur, the episode of CIS in that individual might be considered the first episode of relapsing-remitting MS (RRMS), although definitive clinical diagnosis does not occur until after the second demyelinating event. In RRMS, peripheral blood cells could reflect either the acute inflammation that causes damage to the central nervous system (CNS), or between attacks, they may signal the reparative activity occurring that temporarily resolves the demyelination and symptoms, driven by leukocytes with suppressive activities. Despite this, the phenotypes of peripheral blood cells observed between magnetic resonance imaging (MRI)-diagnosed attacks are not well understood, and their relationships with ongoing disease-specific processes are unclear.

Bi-directional movement of leukocytes is observed between the CNS and the cervical lymph nodes in MS [2], which may explain why MS therapies directed peripherally can reduce CNS inflammation. An investigation of circulating cells at the time of clinically-evident disease activity may therefore give clues to disease pathogenesis as well as provide insight into potential cellular targets for treatment to prevent future demyelinating events. In this study, peripheral blood samples from individuals with CIS (recruited at baseline into an interventional trial with narrowband UV-B phototherapy [3]) were analysed and compared with peripheral blood samples taken from healthy controls. In addition, blood samples taken from people with other acute demyelinating conditions (and recent clinically-evident disease) were included to provide evidence as to whether changes observed were generally associated with MRI demyelination or were specific to CIS at high risk of conversion to MS. We sought to define immunological characteristics specific to cells from individuals with CIS that would, in turn, provide evidence about the pathogenesis of early MS, before definitive diagnosis based on a second demyelinating event. We found that when time since MRI diagnosis and clinical presentation was accounted for in the CIS group, changes to cell subset frequencies in sampled blood emerged that differentiated those with recent diagnosis from those with a delay since diagnosis. These findings indicate the importance of considering time since the clinical event when conducting immunological assessments in CIS.

## 2. Results

### 2.1. Study Participant Demographics

Participants in the healthy control (HC), CIS and other demyelinating conditions (ODC) groups were not significantly different in age or sex (Table 1). However, for many CIS patients compared to those with ODC, a significantly longer time had elapsed between onset of symptoms and diagnostic MRI, and between diagnostic MRI and blood donation. Despite this trend, six people with CIS had significantly shorter periods between symptom onset, diagnostic MRI and blood sampling than other CIS patients, as shown in Table 1. As the time of symptom onset can be subjectively recalled, the date of diagnostic MRI provided the most objective measure for time since start of disease and was used in subsequent analyses.

### 2.2. Peripheral Blood Mononuculear Cell (PBMC) Subset Frequencies in Different Clinical Groups

To investigate whether we could find a CIS-specific signature, we initially compared the frequency of peripheral blood mononuclear cell (PBMC) subsets in all people with CIS to those with ODC and HC (Table A1). The ODC group had its own signature, namely increased CD1c+ B cells and decreased non-classical monocytes as a proportion of all PBMC. In blood samples from people with CIS compared to HC, there were significantly increased frequencies of transitional B cells (IgD+CD27−CD24hiCD38hi B cells) as a percent of B cells, and CD141+ DCs as a percent of DCs. However, as demonstrated in Table 1, the CIS group was heterogeneous in time since symptom onset and in relation to diagnostic MRI. We could not determine whether these results (changes in transitional B cells and CD141+ DCs) were a signature specific to CIS, or were influenced by the variable of time between MRI and blood draw, and so this variable was included in all further analyses. The CIS participants clearly separated into two groups according to the time between diagnostic MRI and blood sampling (Table 1). In one group, the blood sample was taken within 14 days (*n* = 6) of diagnostic MRI (hereafter referred to as “early CIS”), while in the other group, blood was collected ≥27 days after their diagnostic MRI (hereafter referred to as “late CIS”; *n* = 12). The median times since reported symptom onset at the time of blood sampling for the two groups were 13 and 65 days, respectively. For the ODC group, all blood samples were collected within 20 days of diagnostic MRI.

**Figure 1:**
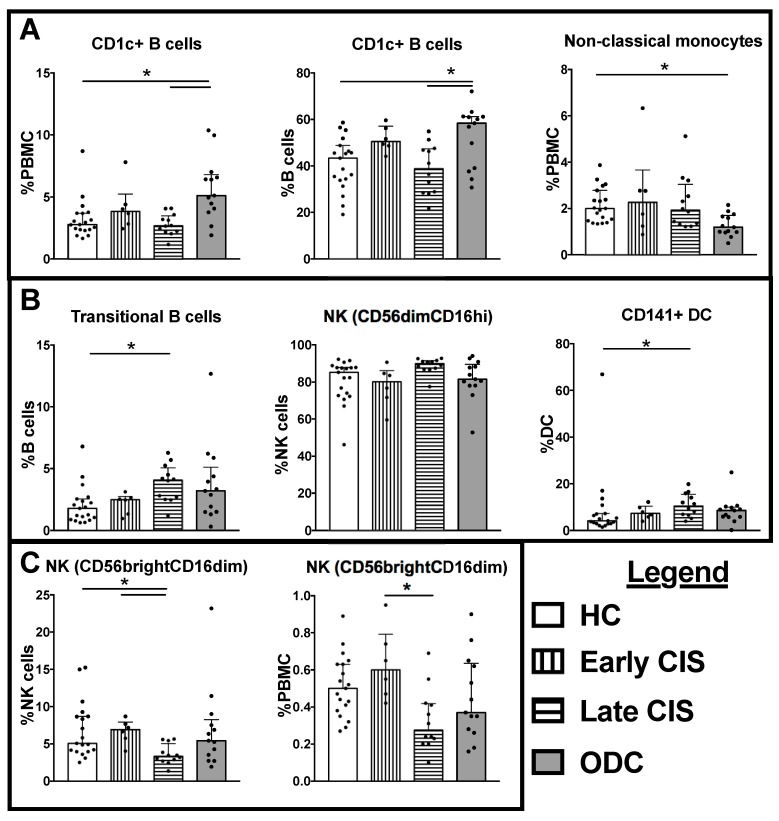
Cell frequencies significantly different between healthy controls (HC), other demyelinating conditions (ODC), early cliniclaly isolated syndrome (CIS) and late CIS. (**A**) Cell types that were significantly altered compared with HC in the ODC group; (**B**) Cell types that were significantly increased from HC in the late CIS group; (**C**) Cell types that were significantly decreased from HC or early CIS in the late CIS group. Individual data are shown in addition to median and interquartile range, indicated by the bar graph and error bars. Significant differences between groups in Kruskal Wallis tests with Bonferroni corrected post-tests are indicated by lines with asterisks. CD56dimCD16hi NK cell frequencies were significantly different in Kruskal Wallis test, but the post-test was not significant between groups.

There were no detectable differences between the four groups (HC, ODC, and two CIS groups) in total monocytes, total DCs, total B cells or total NK cells as a frequency of PBMCs (Table A2). However, when investigating subsets of these cell types, alterations in several NK, B cell and DC subsets in samples from the late CIS individuals were observed, shown in Figure 1 and Table A2. In particular, the late CIS group had significantly lower frequencies of CD56brightCD16loNK cells (% NK cells; Figure 1) compared with early CIS or HC patients.

**Figure 2:**
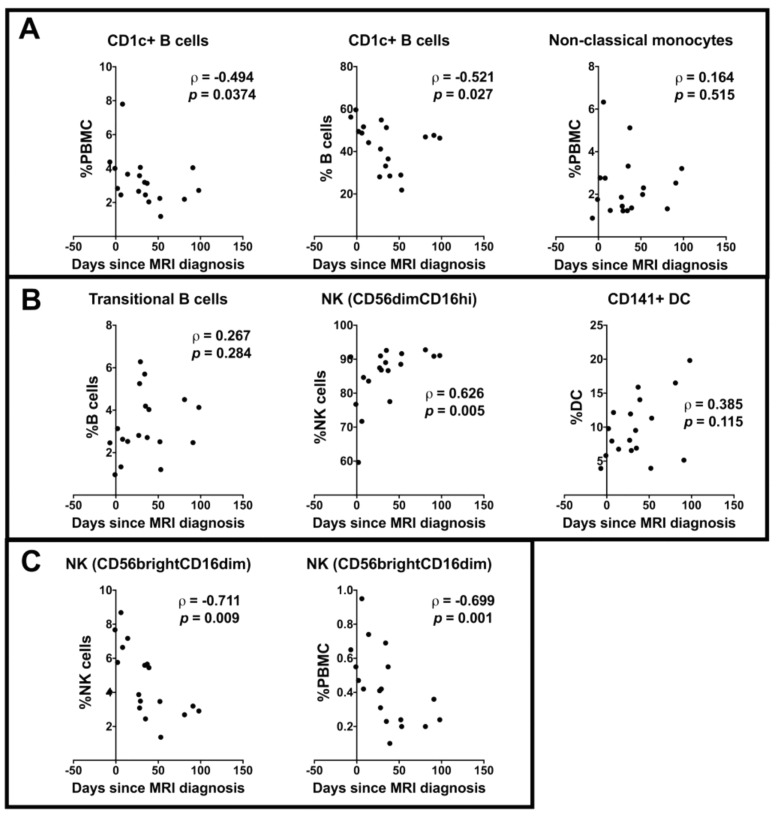
Correlations between time since diagnostic magnetic resonance imaging (MRI) and cell subsets in CIS previously shown to be significantly different to HC in Kruskal Wallis tests. (**A**) Cell types that were significantly altered compared with HC in ODC; (**B**) Cell types that were significantly increased from HC in the late CIS group; (**C**) Cell types that were significantly decreased from HC or early CIS in the late CIS group. Correlations are shown by ρ and *p* values from Spearman correlation tests.

Cell subsets that were significantly different between HC and either of the two CIS sampling groups in the previous analyses (Figure 1) were further investigated in the CIS participants in relation to time since diagnostic MRI, considered as a continuous variable. There was no correlation between the time since diagnostic MRI and the frequencies of transitional B cells, CD141+ DCs or non-classical monocytes (Figure 2). However, a significant negative or positive correlation with days since MRI was observed for CD56bright NK cells, CD56dim NK cells, and CD1c+ B cells in the samples from those with CIS.

## 3. Discussion

This study aimed to identify a CIS-specific PBMC signature that provided clues about dysfunctional immune cells early in the RRMS disease course. People with CIS compared with HC had a peripheral blood signature characterised by significantly increased transitional B cells and CD141+ DCs as a frequency of B cells and DCs, respectively. These changes were not correlated with time since diagnostic MRI, nor were they associated with ODC. However, time-dependent changes in CD1c+ B cells and CD56brightCD16lo NK cell subsets in blood from CIS participants were detected. These data suggest that the timing of the blood sample with reference to symptom onset and diagnostic MRI in the clinical course of CIS is an important factor to consider when interpreting findings for CIS. Potentially, the increases in CD1c+ B cells and CD56bright NK cells we observed after diagnosis of CIS would have prognostic value if these cell frequencies increase proximal to the development of new demyelinating lesions.

Peripheral blood collected in late CIS participants had significantly decreased frequencies of CD56bright NK cells compared with HC, suggesting that there are dynamic changes in this subset in blood during the course of an episode of CIS that may be related to CNS changes. This cell signature was not seen when all CIS patients were initially combined as a single group and compared with HC or ODC. Blood samples collected in the early CIS group displayed a trend toward increased frequencies of these cells compared with HC and displayed significant increases compared with blood cells from late CIS. This finding was reinforced by the negative correlation between CD56bright NK cell frequencies and time since MRI across all CIS blood samples. Our results support previous findings that peripheral blood from people with MS has fewer CD56bright NK cells than HC [4]. Other findings have been reported; CD56bright NK cells were increased in blood from people with RRMS with no disease-associated MRI changes over 2.4 years [5]. CD56 bright NK cells can have multiple functions, including production of cytokines such as IFN-γ, IL-10 and TNF-β, as well as cytotoxic and regulatory activities in T cell responses [5,6]. CD56bright NK cells are reported to be more concentrated in the cerebrospinal fluid (CSF) of people with MS compared with blood from the same donor [7] with defective capacity to inhibit autologous T cells [8]. These cells respond to CCL19 and CCL21 chemokine gradients via CCR7 [9]; chemokines that are upregulated in active brain lesions in chronic relapsing experimental autoimmune encephalomyelitis (EAE) and MS [10,11]. Differences observed in CD56bright NK cell frequencies in this study associated with time since the clinical event in CIS could therefore reflect the immunological activity of brain lesions at the time of blood sampling. We can only speculate whether the situation in the CNS mimics the peripheral blood. However, our observations suggest that migration of CD56bright NK cells through peripheral blood from bone marrow or lymphoid tissue to the site of demyelination occurs early in CIS, and then peripheral blood frequencies of these cells may normalise as the CNS inflammation resolves. Overall, our data support the hypothesis that NK cells are important in the immune changes occurring in CIS, even prior to development of clinically confirmed MS.

Transitional B cells were significantly increased in frequency as a percent of B cells in blood sampled from people with CIS compared with HC. These (and other B cells) respond to the cytokine B cell activating factor of the TNF family (BAFF), which stimulates their proliferation and differentiation [12]. Overexpression of BAFF has been demonstrated in MS lesions; however, atacicept, which was developed to target BAFF for this reason, unexpectedly worsened disease in a human trial [13]. Conversely, a recent study of several current immunomodulatory treatments showed that following administration of IFN-β and fingolimod, an increase in transitional B cells was observed in peripheral blood, associated with increased BAFF levels in plasma [14]. Together, these data suggest that the increased transitional B cells observed in CIS could represent both pathogenic and regulatory subpopulations. That is, either pathogenic transitional B cells respond to the increased BAFF signalling from the CNS and contribute to inflammation, or increased regulatory transitional B cell output from the bone marrow reflects a suppressive response to the inflammation, or both. Since transitional B cells can contain B regulatory cells (Bregs) [15], more markers are needed to determine whether these expanded populations of “transitional” B cells in CIS contain more than one functional population (e.g., identified by IL-10 or IgG4 production), since this could not be determined from our data.

Two cell subsets differentiated ODC from the early CIS group. The first cell subset was the CD56bright NK cell population, which was not different in ODC compared with HC despite differences in frequency in the CIS population. The second differentiating cell type was the non-classical monocyte (CD16+); this was significantly decreased in ODC compared with HC but not in CIS. This finding is supported by recent publications analysing CD16+ monocytes and their gene expression in peripheral blood and the CSF of people with MS [16,17]. CD16+ monocytes from MS patients tend to have increased surface expression of activation markers and respond more robustly with inflammatory cytokine production to in vitro stimulation compared with cells from healthy controls [18,19]. Unless the similarity between HC and CIS is an effect of the small sample size of our early CIS group, these findings suggest that CIS is immunologically distinct from ODC (including MS) in their capacity for immune cells to stabilise to “normal” levels after a demyelinating event in the CNS. Potentially, the decrease of CD16+ monocytes seen in ODC is a result of the changes that occur with repeated demyelination events in the MS group, but this could not be determined in our study.

We have shown, for the first time, that patients who gave blood at times closer to their symptom onset and diagnostic MRI had higher levels of CD1c+ B cells in their peripheral blood compared with HC. This was not apparent for people with late CIS. CD1c on B cells is responsible for presenting lipid antigens to T cells, including self-antigens, and the responding self-reactive T cells contribute to autoimmune diseases such as rheumatoid arthritis [20]. Structural chemistry indicates that the lipids likely to bind to CD1c include cholesterol esters, potentially including MS-relevant molecules such as sphingolipids [20]. Therefore, the increased frequency of CD1c+ B cells may indicate that they have a pathogenic role; in the context of an open blood brain barrier in acute demyelination, the ability of B cells to take up neural antigens and act as antigen-presenting cells to T cells to induce anti-myelin responses may contribute to progression of disease. In this study, inclusion of time since diagnostic MRI as a variable in people with ODC and CIS allowed confirmation of changes that may occur closer to the clinical event. The benefit of this approach was clearly demonstrated in reference to the CD1c+ B cell population, a cell that may present a diagnostic marker of CNS demyelination that can be related to MRI findings.

Given that immune-modulating therapies for demyelinating diseases are often delivered peripherally with beneficial CNS effects, as well as the bi-directional movement of cells between the CNS and periphery, the profile of blood cells may reflect the disease course in the CNS. Although ideally one would biopsy the demyelinating lesion or sample the CSF in patients with demyelinating disease, these are far more invasive procedures than blood collection, with CSF having the limitation of lower cell counts [21]. Consequently, we chose to study more accessible peripheral blood, which is much more frequently accessible with abundant leukocytes for characterisation. While it has been reported that some cell frequencies correlate between the CSF and blood, other cells frequencies may be independent in each compartment [7]. Despite this limitation, the current study shows the relevance of time-dependent peripheral blood changes in CIS, and the importance of careful interpretation of cell changes in peripheral blood with reference to those of healthy controls. Our findings suggested that at blood sampling times closer to the time of clinical presentation and diagnostic MRI, alterations to the cell frequency profile compared with HC may reflect responses aligned with the demyelinating event in the CNS. However, at later sampling time points, cell profiles may reflect ongoing pathogenic and/or reparative processes. In this study, the two CIS groups separated easily according to time between diagnostic MRI and blood sampling. The smaller group of six early CIS patients was similar to the ODC participants in time from blood draw to diagnostic MRI and symptom onset, and while these patients had similarities in many cell types, they could be distinguished from ODC based on frequencies of some innate immune cells (NK cells and non-classical monocytes). Given that all ODC samples were collected early in the disease course, it was not possible to assess the effect of time to sampling from MRI in ODC, and this could be the subject of future investigations.

## 4. Materials and Methods

### 4.1. Study Participants

Individuals presenting with a first demyelinating event at <120 days post-MRI diagnosis of CIS were included (*n* = 18). In addition, 19 HC, and 13 people with other demyelinating conditions (ODC; seven with acute optic neuritis, one with clinically isolated myelitis, all with an isolated appropriate MRI abnormality, and five with acute relapse of definite MS according to the current consensus McDonald criteria [22]) were recruited. None of the study participants had received disease-modifying treatments (including steroids) for at least one month prior to blood sampling. Pregnant women were excluded. CIS was diagnosed from demyelination on MRI according to PatyA or PatyB criteria, whereas those with optic neuritis and clinically isolated myelitis included in ODC had MRI changes consistent with the presenting demyelinating lesion but not satisfying PatyA or PatyB criteria.

Clinical data including age, sex, reported date of symptom onset, and MRI date were collected for all participants. This study was approved by the University of Western Australia Human Research Ethics Committee (2014-02-083, approved 22 April 2014) and Sir Charles Gairdner Hospital Human Research Ethics Committee (2006-073, approved 27 September 2006), and all participants provided written consent before any study procedures were performed.

### 4.2. Flow Cytometry

PBMCs were isolated from fresh heparinized blood collected in lithium heparin vacutainers (BD) using a Lymphoprep (Axis-Shield, Oslo, Norway) density separation gradient. Freshly isolated PBMCs were stained and examined for a range of cell types using antibodies to surface markers characteristic of B, NK, monocyte, and dendritic cell (DC) subsets.

PBMCs were stained by incubating 10^6^ cells at 4 °C in 100 µL of phosphate buffered saline (PBS) with the following mouse anti-human antibodies: FITC conjugated anti-CD45 (clone HI30), PE conjugated anti-CD19 (HIB19), PE-CF594 conjugated anti-CD24 (ML5), PE-Cy7 conjugated anti-IgD (IA6-2), APC-H7 conjugated anti-CD20 (2H7), BV421 conjugated anti-CD38 (HIT2), BV510 conjugated anti-CD27 (L128), FITC conjugated anti-CD303 (AC144), PE conjugated anti-CD1c (AD5-8E7), PE-CF594 conjugated anti-CD56 (B159), PerCP-Cy5.5 conjugated anti-CD19 (HIB19), PerCP-Cy5.5 conjugated anti-CD20 (2H7), PE-Cy7 conjugated anti-cD14 (M5E2), Alexa Fluor 700 conjugated anti-CD3 (UCHT1), APC-H7 conjugated anti-CD16 (3G8), BV421 conjugated anti-CD57 (NK-1), and BV711 conjugated anti-CD141 (1A4). All antibodies were purchased from BD (North Ryde, NSW, Australia), except for anti-CD303 and anti-CD1c, which were purchased from Miltenyi Biotech (Macquarie Park, NSW, Australia).

After incubation, cells were washed with sterile PBS before resuspension in PBS for analysis. Data were acquired using the BD LSRFortessa flow cytometer instrument in combination with FACSDiva software (Version 8.0.1, BD Biosciences, Franklin Lakes, NJ, USA) and analysed using FlowJo v10 (Tree Star, Ashland, OR, USA). Approximately 200,000–500,000 events were acquired for each sample.

**Figure 3:**
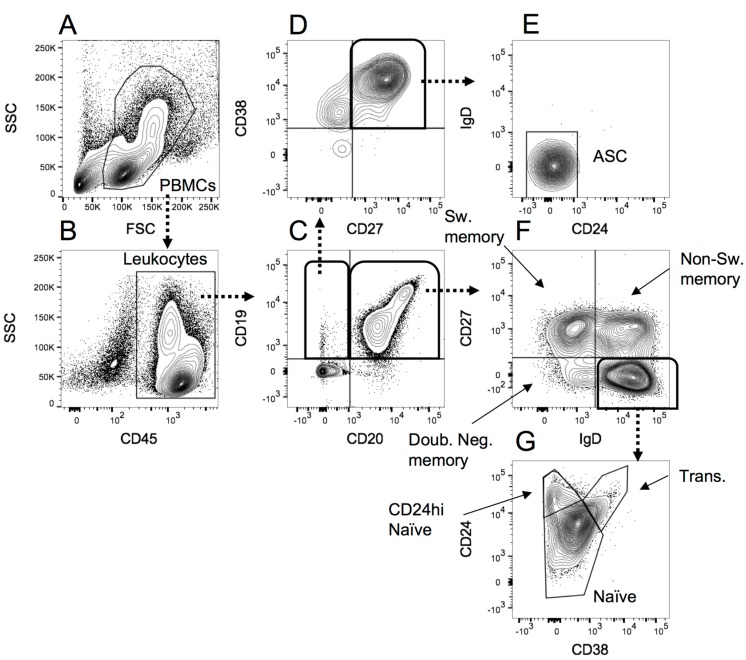
The gating strategy applied to peripheral blood mononuclear cells (PBMCs) analysed for B cell subsets. (**A**) PBMCs were selected according to forward scatter (FSC)/side scatter (SSC) profile; (**B**) Leukocytes were identified based on CD45 expression; (**C**) CD19+CD20− B cells were separated from double positive and CD19− cells; (**D**) CD19+CD20− B cells that were positive for CD27 and CD38 were selected as probable antibody secreting cells; (**E**) Identification of antibody secreting cells (ASC) was confirmed from negative expression of CD24 and IgD; (**F**) Double positive B cells identified in C were separated into switched memory, non-switched memory and double negative memory B cells using CD27 and IgD expression; (**G**) IgD+CD27− cells in **F** were separated into naïve B cells (all CD38−/low), CD24hi naïve and transitional B cells using CD38 and CD24 expression.

**Figure 4:**
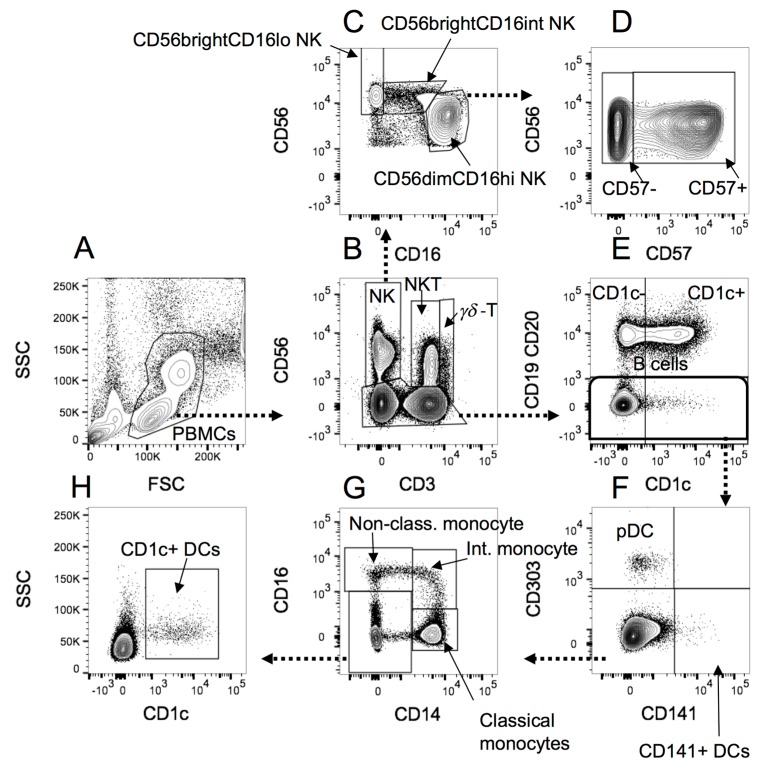
The gating strategy applied to PBMCs analysed for monocytes, NK cells and DCs. (**A**) PBMCs were selected according to the forward scatter (FSC)/side scatter (SSC) profile; (**B**) NK cells, NKT and γδ-T cells were separated from CD56− cells, and from one another according to CD3 staining; (**C**) NK cells gated in B were separated into CD56brightCD16lo, CD56brightCD16int and CD56dim cells, according to CD16 and CD56 staining; (**D**) CD56dim NK cells were further separated into CD57+ or CD57− according to CD57 expression; (**E**) B cells were separated from non-B cells in the non-NK, NKT or γδ-T cell CD56- category identified in B. B cells were also divided into CD1c+ or CD1c− populations; (**F**) Non-B cells identified in E were divided into CD141+ myeloid DCs (mDC2), CD303+ plasmacytoid DCs (pDCs), or other cells. (**G**) Cells negative for CD141 and CD303 staining in F were separated based on CD16 and CD14 expression into classical (CD14hiCD16−), intermediate (CD14+CD16+) and non-classical (CD14loCD16+) monocytes (reference [23]), as well as CD14−CD16− cell types; (**H**) The CD14−CD16− cells identified in G were gated on CD1c+ to identify CD1c+myeloid DCs (mDC1).

The B cell gating was performed as shown in Figure 3, and the monocytes, NK cells and DCs gated as shown in Figure 4 below.

### 4.3. Statistical Analyses

Continuous data were tested for normality using a Shapiro-Wilk test. Non-parametric correlation was described between continuous variables including time since MRI and flow cytometry frequency data using Spearman’s rho (ρ). The differences between the HC, CIS and ODC groups were tested using a Kruskal-Wallis test using Bonferroni’s correction in the post-test (where appropriate) for continuous variables, and a Chi-squared test for categorical variables including sex. Cells identified in flow cytometry were analysed and presented as a frequency of PBMCs and as a frequency of B cells, monocytes, dendritic cells or NK cells, where relevant. The alpha value for all tests was set to 0.05.

## 5. Conclusions

It is clear from our analytical approach that while it is common to report whether blood studied was sampled within one month of immunomodulatory therapies, it is equally important to state when blood was taken relative to the time of clinical presentation and diagnosis due to the dynamic cell activities observed in CIS. Prognostic markers, easily obtained from peripheral blood and assessed using flow cytometry, would be valuable to guide clinical care decisions. Therefore, the association between cell frequencies at the time of this first symptomatic demyelinating event and clinical and MRI outcomes at later time points will be the subject of future investigations upon trial completion.

## Figures and Tables

**Table 1:** Demographic data for the study cohort.

Clinical Characteristics	Healthy Controls (HC) (*n* = 19)	Other Demyelinating Conditions (ODC) (*n* = 13)	Early Clinically Isolated Syndrome (CIS) (≤14 Days Post-MRI) (*n* = 6)	Late Clinically Isolated Syndrome (CIS) (≥27 Days Post-MRI) (*n* = 12)	*p* Value
Age (median (range))	41 (22, 61)	34 (18, 75)	35 (29, 49)	42 (23, 54)	0.444
Sex (F), n (%)	12 (63%)	8 (62%)	3 (50%)	9 (75%)	0.758
Days from MRI diagnosis to blood sampling (median (range))	n/a	4 (−2, 20) ^a^	4 (−7, 14) ^b^	38 (27, 98) ^a,b^	**<0.001**
Days from reported symptom onset to blood sampling (median (range))	n/a	10 (4, 27) ^a^	13 (6, 31) ^b^	65 (39, 137) ^a,b^	**<0.001**
Days from symptom onset to diagnostic MRI (median (range))	n/a	6 (0, 27) ^a^	11.5 (7,17)	26 (2, 101) ^a^	**0.004**

Legend: n/a = not applicable. Letter superscripts indicate groups that were significantly different from one another in post-tests. Bolded formatting for *p* values indicate values that were considered statistically significant.

## Abbreviations

APC	Allophycocyanin
BAFF	B cell activating factor
BV	Brilliant Violet
CCL19	Chemokine ligand 19
CCL21	Chemokine ligand 21
CCR7	Chemokine receptor 7
CIS	Clinically isolated syndrome
CNS	Central nervous system
CSF	Cerebrospinal fluid
DC	Dendritic cell
EAE	Experimental autoimmune encephalomyelitis
FITC	Fluorescein isothiocyanate
HC	Healthy control
IFN-γ	Interferon gamma
IgG4	Immunoglobulin G subclass 4
IL-10	Interleukin 10
ODC	Other demyelinating conditions
PBMC	Peripheral blood mononuclear cell
PE	Phycoerythrin
PBS	Phosphate buffered saline
MRI	Magnetic resonance imaging
MS	Multiple sclerosis
NK	Natural killer
RRMS	Relapsing-remitting multiple sclerosis
TNF-β	Tumor necrosis factor beta
UV-B	Ultraviolet B

## Appendix A

**Table A1:** Frequencies of cell subsets in healthy controls (HC), other demyelinating conditions (ODC), and CIS analysed by flow cytometry according to the percentage of total PBMCs, and where relevant, percentage of total B cells, monocytes, NK cells or DCs.

Cell Type	HC (*n* = 19)		ODC (*n* = 13)		CIS (*n* = 18)		*p* Value
	Median	IQR	Median	IQR	Median	IQR	
%PBMC							
γδ−Τ cells	0.43	0.28–0.76	0.61	0.23–1	0.38	0.29–0.58	0.891
NKT cells	2.21	1.33–4.1	2.32	1.38–2.72	1.92	1.14–2.62	0.519
Total B cells	8.55	5.71–10.56	7.86	7.06–10.89	7.55	5.41–9.33	0.476
Total DCs	0.86	0.68–1.09	0.74	0.51–0.87	0.92	0.77–1.05	0.165
Total monocytes	14.74	10.87–15.71	9.01	5.33–18.1	15.90	11.08–17.95	0.093
Total NKs	9.59	6.44–11.46	7.72	4.94–9.79	9.66	7.68–11.39	0.374
B cells (% PBMC)							
Antibody secreting cells	0.04	0.02–0.06	0.06	0.03–0.13	0.04	0.03–0.12	0.479
CD1c− B cells	4.43	3.03–6.48	4.26	3.82–5.85	4.34	2.89–5.45	0.687
CD1c+ B cells	**2.77 ^a^**	**2.37–3.68**	**5.11 ^a,b^**	**4.06–6.56**	**2.98 ^b^**	**2.44–4**	**0.003**
CD24hi CD38- B cells	0.35	0.28–0.5	0.50	0.23–0.55	0.47	0.44–0.67	0.344
CD27+ B cells	3.00	2.06–3.32	2.37	1.67–3.82	2.11	2–3.12	0.628
Double negative memory B cells	0.24	0.15–0.43	0.33	0.23–0.39	0.28	0.21–0.49	0.41
Naïve B cells	4.49	2.58–6.21	4.94	3.81–5.33	4.14	2.57–6.41	0.745
Non-switched memory B cells	1.39	1.06–2.01	0.95	0.5–1.85	1.18	0.94–1.48	0.263
Switched memory B cells	1.08	0.75–1.62	1.34	0.98–1.97	1.13	0.84–1.6	0.516
Total memory B cells	3.17	2.23–3.79	2.69	1.88–4.17	2.62	2.24–3.3	0.746
Transitional B cells	0.11	0.05–0.22	0.26	0.18–0.38	0.22	0.14–0.34	0.086
Monocytes (%PBMC)							
Classical monocytes	11.85	8.7–13.1	7.81	3.91–15.46	12.11	9.19–16.13	0.215
Intermediate monocytes	0.65	0.53–1.06	0.47	0.28–0.7	0.85	0.53–1.03	0.101
Non-classical monocytes	**1.99 ^a^**	**1.46–2.77**	**1.18 ^a,b^**	**0.98–1.64**	**1.92 ^b^**	**1.32–2.77**	**0.003**
NK cells (%PBMC)							
CD56dimCD16hi NK cells	7.61	4.68–10.5	6.76	3.85–8.21	8.31	6.46–10.35	0.397
CD56dimCD16hiCD57- NK cells	1.91	1.32–2.96	1.68	1.38–2.33	2.03	1.78–3.07	0.436
CD56dimCD16hiCD57+ NK cells	5.46	3.35–7.12	4.63	2.06–5.55	5.51	4.35–6.74	0.568
CD56brightCD16int NK cells	0.32	0.22–0.6	0.33	0.22–0.59	0.38	0.27–0.49	0.885
CD56brightCD16lo NK cells	0.49	0.37–0.62	0.36	0.28–0.61	0.41	0.23–0.55	0.333
DCs (%PBMC)							
mDC1	0.44	0.28–0.51	0.32	0.25–0.43	0.36	0.3–0.51	0.428
mDC2	0.03	0.01–0.08	0.06	0.02–0.07	0.06	0.04–0.12	0.094
pDC	0.37	0.33–0.5	0.23	0.17–0.39	0.39	0.33–0.5	0.086
B cells (%B cells)							
Antibody secreting cells	0.49	0.27–1	0.52	0.39–1.17	0.67	0.36–1.55	0.598
CD1c− B cells	**56.58 ^a^**	**51.16–66.14**	**41.63 ^a^**	**38.86–60.92**	**53.42**	**48.75–66.84**	**0.018**
CD1c+ B cells	**43.41 ^a^**	**33.85–48.83**	**58.36 ^a^**	**39.07–61.13**	**46.57**	**33.15–51.24**	**0.018**
CD24hiCD38−IgD+CD27− B cells	5.82	3.08–9	5.40	3.65–6.85	6.38	4.23–11.68	0.415
CD27− B cells	60.67	56.68–69.43	66.37	60.15–75.44	60.67	56.7–71.89	0.478
CD27+ B cells	37.72	30.02–39.2	32.21	21.33–39.05	29.96	26.77–39.46	0.532
Double negative memory B cells	3.49	2.18–4.58	4.08	3.33–4.67	4.73	3.02–5.89	0.171
Naive B cells	57.66	50.19–62.89	58.68	52.92–66.86	54.05	47.85–66.53	0.588
Non-switched memory B cells	21.18	11.96–24.42	14.67	4.52–19.79	14.11	11.35–22.55	0.316
Switched memory B cells	16.53	11.47–19.89	17.22	14.54–17.62	16.22	13.02–18.88	0.964
Total memory B cells	39.82	34.33–45.48	36.19	25.87–42.64	35.85	30.1–45.16	0.677
Transitional B cells	**1.79 ^a^**	**0.88–2.54**	**3.20**	**1.5–4.37**	**2.75 ^a^**	**2.47–4.19**	**0.016**
Monocytes (% Monocytes)							
All CD16+ monocytes	21.54	17.11–25.82	22.02	13.28–28.39	18.82	16.76–27.1	0.689
Classical monocytes	78.45	74.17–82.88	77.97	71.6–86.71	77.38	71.28–83.23	0.937
Intermediate monocytes	5.68	4.48–7.25	5.54	4.74–7.39	5.48	4.39–7.23	0.962
Non-classical monocytes	15.12	12.19–17.98	10.59	8.23–22.23	11.57	9.34–13.76	0.27
NK cells (% NK cells)							
All CD57− NK cells	40.53	34.44–46.76	44.66	34.81–54.67	35.69	27.45–43.83	0.394
All CD57+ NK cells	59.46	52.95–65.44	55.32	45.32–65.18	64.30	56.13–72.53	0.36
CD56dimCD16hi NK cells	85.24	72.69–87.87	81.50	78.06–88.26	87.98	83.56–90.98	0.304
CD56dimCD16hiCD57− NK cells	24.74	15.97–28.89	25.18	19.05–29.44	20.59	17.66–27.44	0.801
CD56dimCD16hiCD57+ NK cells	55.69	48.7–62.41	54.77	43.84–64.84	62.19	53.14–73.11	0.367
CD56brightCD16lo NK cells	5.07	3.95–8.67	5.40	3.52–7.51	3.95	3.09–5.76	0.168
CD56brightCD16int NK cells	5.32	2.26–7.36	4.73	3.38–6.83	3.34	2.88–5.1	0.504
DC (%DC)							
All mDC	57.10	49.51–62.99	53.52	43.38–68.46	52.21	46.24–58.39	0.756
mDC1	50.16	40.29–55.09	47.59	30.78–58.68	43.41	37.59–50.22	0.513
mDC2	**4.12 ^a^**	**2.89–7.22**	**8.57**	**5.82–9.78**	**8.79 ^a^**	**6.56–12.16**	**0.026**
pDC	42.89	37–50.48	46.47	31.53–56.61	47.78	41.6–53.75	0.756

Groups shown were compared using a Kruskal Wallis test; significant results are indicated by bold *p* values and shaded background. Letter superscripts indicate groups that were significantly different from each other in post-tests with Bonferroni’s correction. IQR = interquartile range.

## Appendix B

**Table A2:** Frequencies of cell subsets in healthy controls, other demyelinating conditions (ODC), early CIS and late CIS analysed by flow cytometry according to the percentage of total PBMCs, and where relevant, percentage of total B cells, monocytes, NK cells or DCs.

Cell Type	Healthy Controls (*n* = 19)		ODC (*n* = 13)		Early CIS [≤14 Days Post MRI (*n* = 6)]		Late CIS [≥27 Days Post MRI (*n* = 12)]		*p* Value
	Median	IQR	Median	IQR	Median	IQR	Median	IQR	
%PBMC									
γδ-T	0.43	0.28–0.76	0.61	0.23–1	0.36	0.24–0.57	0.43	0.33–0.95	0.845
NKT cells	2.21	1.33–4.1	2.32	1.38–2.72	1.79	1.14–2.49	1.96	1.24–2.66	0.711
Total B cells	8.55	5.71–10.56	7.86	7.06–10.89	6.19	4.87–7.32	8.99	6.71–9.55	0.429
Total DC	0.86	0.68–1.09	0.74	0.51–0.87	0.92	0.71–0.99	0.90	0.78–1.07	0.287
Total monocytes	14.74	10.87–15.71	9.01	5.33–18.1	15.90	11.01–18.46	15.90	11.26–17.93	0.190
Total NK cells	9.59	6.44–11.46	7.72	4.94–9.79	8.91	7.68–10	9.74	7.64–12.16	0.495
B cells (%PBMC)									
Antibody secreting cells	0.04	0.02–0.06	0.06	0.03–0.13	0.03	0.03–0.04	0.08	0.02–0.16	0.327
CD1c− B cells	4.43	3.03–6.48	4.26	3.82–5.85	3.15	2.71–4.63	4.78	3.24–5.47	0.628
CD1c+ B cells	2.77 ^a^	2.37–3.68	5.11 ^a,b^	4.06–6.56	3.83	2.82–4.38	2.68 ^b^	2.21–3.38	**0.003**
CD24hi Naïve B cells	0.35	0.28–0.5	0.50	0.23–0.55	0.53	0.46–0.72	0.46	0.34–0.63	0.376
Double negative memory B cells	0.24	0.15–0.43	0.33	0.23–0.39	0.27	0.22–0.36	0.31	0.2–0.54	0.486
Naïve B cells	4.49	2.58–6.21	4.94	3.81–5.33	2.58	2.56–3.76	5.26	2.88–6.43	0.418
Non-switched memory B cells	1.39	1.06–2.01	0.95	0.5–1.85	1.33	1.08–1.97	1.13	0.91–1.32	0.315
Switched memory B cells	1.08	0.75–1.62	1.34	0.98–1.97	1.26	0.84–1.85	1.11	0.87–1.51	0.714
Total CD27+ B cells	3.00	2.06–3.32	2.37	1.67–3.82	2.58	1.92–3.34	2.11	2.02–2.8	0.742
Total memory B cells	3.17	2.23–3.79	2.69	1.88–4.17	2.87	2.16–3.74	2.62	2.28–3.17	0.871
Transitional B cells	0.11	0.05–0.22	0.26	0.18–0.38	0.13	0.06–0.18	0.26	0.19–0.43	**0.036 (post-test NS)**
Monocytes (%PBMC)									
Classical monocytes	11.85	8.7–13.1	7.81	3.91–15.46	12.25	7.82–17.1	12.11	9.24–15.49	0.378
Intermediate monocytes	0.65	0.53–1.06	0.47	0.28–0.7	0.73	0.45–1.78	0.85	0.61–1	0.188
Non-classical monocytes	1.99 ^a^	1.46–2.77	1.18 ^a^	0.98–1.64	2.26	1.23–2.77	1.92	1.34–2.86	**0.009**
NK (%PBMC)									
CD56dimCD16hi NK cells	7.61	4.68–10.5	6.76	3.85–8.21	7.15	6.04–8.46	8.76	6.77–10.7	0.391
CD56dimCD16hiCD57− NK cells	1.91	1.32–2.96	1.68	1.38–2.33	2.03	1.96–3.07	2.09	1.64–2.87	0.620
CD56dimCD16hiCD57+ NK cells	5.46	3.35–7.12	4.63	2.06–5.55	4.85	3.76–5.81	6.50	4.42–8.73	0.510
CD56brightCD16int NK cells	0.32	0.22–0.6	0.33	0.22–0.59	0.46	0.4–0.69	0.29	0.23–0.45	0.284
CD56brightCD16lo NK cells	0.49	0.37–0.62	0.36	0.28–0.61	0.59 ^a^	0.46–0.73	0.27 ^a^	0.21–0.41	**0.012**
DCs (%PBMC)									
mDC1	0.44	0.28–0.51	0.32	0.25–0.43	0.30	0.28–0.34	0.43	0.33–0.55	0.283
mDC2	0.03	0.01–0.08	0.06	0.02–0.07	0.05	0.03–0.06	0.09	0.06–0.15	**0.073**
pDC	0.37	0.33–0.5	0.23	0.17–0.39	0.34	0.31–0.36	0.45	0.36–0.71	**0.045 (post-test NS)**
B cells (%B cells)									
Antibody secreting cells	0.49	0.27–1	0.52	0.39–1.17	0.57	0.36–0.7	0.92	0.29–2.19	0.770
CD1c− B cells	56.58 ^a^	51.16–66.14	41.63 ^a,b^	38.86–60.92	49.46	43.81–51.31	61.17 ^b^	52.77–71.3	**0.006**
CD1c+ B cells	43.41 ^a^	33.85–48.83	58.36 ^a,b^	39.07–61.13	50.53	48.68–56.18	38.82 ^b^	28.69–47.22	**0.006**
CD24hi Naïve B cells	5.82	3.08–9	5.40	3.65–6.85	9.50	6.38–13.31	5.66	4.11–10.2	0.338
CD27− B cells	60.67	56.68–69.43	66.37	60.15–75.44	58.04	56.9–62.27	68.60	55.6–73.48	0.413
Double Negative Memory B cells	3.49	2.18–4.58	4.08	3.33–4.67	4.13	3.04–4.98	5.14	2.58–5.96	0.296
Naive B cells	57.66	50.19–62.89	58.68	52.92–66.86	52.05	47.85–55.33	57.84	46.7–66.54	0.585
Non-switched memory B cells	21.18	11.96–24.42	14.67	4.52–19.79	23.92	12.38–26.92	13.04	11.27–17.13	0.147
Switched memory B cells	16.53	11.47–19.89	17.22	14.54–17.62	16.22	14.18–26.17	16.04	11.87–18.32	0.906
Total CD27+ B cells	37.72	30.02–39.2	32.21	21.33–39.05	39.01	36.54–42.13	28.61	24.35–33.44	0.144
Total memory B cells	39.82	34.33–45.48	36.19	25.87–42.64	44.80	40.35–45.25	33.67	29.96–39.25	0.244
Transitional B cells	1.79^a^	0.88–2.54	3.20	1.5–4.37	2.49	1.32–2.62	4.08 ^a^	2.6–4.87	**0.010**
Monocytes (% monocytes)									
All CD16+ monocytes	21.54	17.11–25.82	22.02	13.28–28.39	19.69	13.35–21.42	18.17	16.89–27.21	0.848
Classical monocytes	78.45	74.17–82.88	77.97	71.6–86.71	77.38	70.83–83.11	77.03	71.33–83.46	0.988
Intermediate monocytes	5.68	4.48–7.25	5.54	4.74–7.39	5.48	4.29–5.93	5.63	4.68–7.24	0.953
Non-classical monocytes	15.12	12.19–17.98	10.59	8.23–22.23	13.76	9.05–15.93	11.32	9.59–12.94	0.435
DC (%DC)									
mDC	57.10	49.51–62.99	53.52	43.38–68.46	52.12	50.67–58.05	52.82	44.88–59.06	0.903
mDC1	50.16	40.29–55.09	47.59	30.78–58.68	44.11	40.75–48.26	42.27	37.1–50.34	0.685
mDC2	4.12^a^	2.89–7.22	8.57	5.82–9.78	7.35	5.81–9.78	10.41 ^a^	6.73–14.96	**0.042**
pDC	42.89	37–50.48	46.47	31.53–56.61	47.87	41.94–49.32	47.17	40.93–55.11	0.903
NK (%NK)									
All 57− NK cells	40.53	34.44–46.76	44.66	34.81–54.67	42.00	36.08–44.37	31.45	26.17–43.44	0.219
All CD57+ NK cells	59.46	52.95–65.44	55.32	45.32–65.18	57.94	55.62–63.9	68.39	56.51–73.69	0.203
CD56dimCD16hi CD57− NK cells	24.74	15.97–28.89	25.18	19.05–29.44	25.07	18.44–27.44	20.27	17.2–28.29	0.822
CD56dimCD16hi CD57+ NK cells	55.69	48.7–62.41	54.77	43.84–64.84	54.59	42.58–59.48	67.36	57.13–73.44	0.092
CD56dimCD16hi NK cells	85.24	72.69–87.87	81.50	78.06–88.26	80.14	71.69–84.63	89.95	87.13–91.35	**0.028 (post-test NS)**
CD56brightCD16int NK cells	5.32	2.26–7.36	4.73	3.38–6.83	5.41	2.25–6.98	3.32	2.91–4.17	0.501
CD56brightCD16lo NK cells	5.07 ^a^	3.95–8.67	5.40	3.52–7.51	6.90 ^b^	5.76–7.66	3.33 ^a,b^	2.8–4.65	**0.011**

Groups shown were compared using a Kruskal Wallis test; significant results are indicated by bold *p* values and shaded background. Letter superscripts indicate groups that were significantly different from one other in post-tests with Bonferroni’s correction. IQR = interquartile range; NS = not statistically significant.

## References

[1] Miller D.H., Chard D.T., Ciccarelli O. (2012). Clinically isolated syndromes. Lancet Neurol..

[2] Palanichamy A., Apeltsin L., Kuo T.C., Sirota M., Wang S., Pitts S.J., Sundar P.D., Telman D., Zhao L.Z., Derstine M. (2014). Immunoglobulin class-switched B cells form an active immune axis between CNS and periphery in multiple sclerosis. Sci. Transl. Med..

[3] Hart P.H., Lucas R.M., Booth D.R., Carroll W.M., Nolan D., Cole J.M., Jones A.P., Kermode A.G. (2017). Narrowband UVB phototherapy for Clinically Isolated Syndrome: A trial to deliver the benefits of vitamin D and other UVB-induced molecules. Front. Immunol..

[4] McKay F.C., Gatt P.N., Fewings N., Parnell G.P., Schibeci S.D., Basuki M.A., Powell J.E., Goldinger A., Fabis-Pedrini M.J., Kermode A.G. (2016). The low EOMES/TBX21 molecular phenotype in multiple sclerosis reflects CD56+ cell dysregulation and is affected by immunomodulatory therapies. Clin. Immunol..

[5] Caruana P., Lemmert K., Ribbons K., Lea R., Lechner-Scott J. (2016). Natural killer cell subpopulations are associated with MRI activity in a relapsing-remitting multiple sclerosis patient cohort from Australia. Mult. Scler..

[6] Michel T., Poli A., Cuapio A., Briquemont B., Iserentant G., Ollert M., Zimmer J. (2016). Human CD56bright NK Cells: An Update. J. Immunol..

[7] Han S., Lin Y.C., Wu T., Salgado A.D., Mexhitaj I., Wuest S.C., Romm E., Ohayon J., Goldbach-Mansky R., Vanderver A. (2014). Comprehensive immunophenotyping of cerebrospinal fluid cells in patients with neuroimmunological diseases. J. Immunol..

[8] Laroni A., Armentani E., de Rosbo N.K., Ivaldi F., Marcenaro E., Sivori S., Gandhi R., Weiner H.L., Moretta A., Mancardi G.L. (2016). Dysregulation of regulatory CD56bright NK cells/T cells interactions in multiple sclerosis. J. Autoimmun..

[9] Mehling M., Burgener A.V., Brinkmann V., Bantug G.R., Dimeloe S., Hoenger G., Kappos L., Hess C. (2015). Tissue distribution dynamics of human NK cells inferred from peripheral blood depletion kinetics after sphingosine-1-phosphate receptor blockade. Scand. J. Immunol..

[10] Bielecki B., Jatczak-Pawlik I., Wolinski P., Bednarek A., Glabinski A. (2015). Central nervous system and peripheral expression of CCL19, CCL21 and their receptor CCR7 in experimental model of multiple sclerosis. Arch. Immunol. Ther. Exp..

[11] Krumbholz M., Theil D., Steinmeyer F., Cepok S., Hemmer B., Hofbauer M., Farina C., Derfuss T., Junker A., Arzberger T. (2007). CCL19 is constitutively expressed in the CNS, up-regulated in neuroinflammation, active and also inactive multiple sclerosis lesions. J. Neuroimmunol..

[12] Rowland S.L., Leahy K.F., Halverson R., Torres R.M., Pelanda R. (2010). BAFF-R signaling aids the differentiation of immature B cells into transitional B cells following tonic BCR signaling. J. Immunol..

[13] Kappos L., Hartung H.P., Freedman M.S., Boyko A., Radu E.W., Mikol D.D., Lamarine M., Hyvert Y., Freudensprung U., Plitz T. (2014). Atacicept in multiple sclerosis (ATAMS): A randomised, placebo-controlled, double-blind, phase 2 trial. Lancet Neurol..

[14] Dooley J., Pauwels I., Franckaert D., Smets I., Garcia-Perez J.E., Hilven K., Danso-Abeam D., Terbeek J., Nguyen A.T.L., de Muynck L. (2016). Immunologic profiles of multiple sclerosis treatments reveal shared early B cell alterations. Neurol. Neuroimmunol. Neuroinflamm..

[15] Blair P.A., Noreña L.Y., Flores-Borja F., Rawlings D.J., Isenberg D.A., Ehrenstein M.R., Mauri C. (2010). CD19+CD24hiCD38hi B cells exhibit regulatory capacity in healthy individuals but are functionally impaired in systemic lupus erythematosus patients. Immunity.

[16] Waschbisch A., Schröder S., Schraudner D., Sammet L., Weksler B., Melms A., Pfeifenbring S., Stadelmann C., Schwab S., Linker R.A. (2016). Pivotal role for CD16+ monocytes in immune surveillance of the central nervous system. J. Immunol..

[17] Fewings N.L., Gatt P.N., McKay F.C., Parnell G.P., Schibeci S.D., Edwards J., Basuki M.A., Goldinger A., Fabis-Pedrini M.J., Kermode A.G. (2017). The autoimmune risk gene *ZMIZ1* is a vitamin D responsive marker of a molecular phenotype of multiple sclerosis. J. Autoimmun..

[18] Chuluundorj D., Harding S.A., Abernethy D., La Flamme A.C. (2014). Expansion and preferential activation of the CD14+CD16+ monocyte subset during multiple sclerosis. Immunol. Cell Biol..

[19] Chuluundorj D., Harding S.A., Abernethy D., La Flamme A.C. (2017). Glatiramer acetate treatment normalized the monocyte activation profile in MS patients to that of healthy controls. Immunol. Cell Biol..

[20] Mansour S., Tocheva A.S., Cave-Ayland C., Machelett M.M., Sander B., Lissin N.M., Molloy P.E., Baird M.S., Stubs G., Schroder N.W. (2016). Cholesteryl esters stabilize human CD1c conformations for recognition by self-reactive T cells. Proc. Natl. Acad. Sci. USA.

[21] Longhini A.L.F., von Glehn F., Brandão C.O., de Paula R.F., Pradella F., Moraes A.S., Oliveira E.C., Quispe-Cabanillas J.G., Abreu C.H. (2011). Plasmacytoid dendritic cells are increased in cerebrospinal fluid of untreated patients during multiple sclerosis relapse. J. Neuroinflamm..

[22] Polman C.H., Reingold S.C., Banwell B., Clanet M., Cohen J.A., Filippi M., Fujihara K., Havrdova E., Hutchinson M., Kappos L. (2011). Diagnostic criteria for multiple sclerosis: 2010 revisions to the McDonald criteria. Ann. Neurol..

[23] Ziegler-Heitbrock L., Ancuta P., Crowe S., Dalod M., Grau V., Hart D.N., Leenen P.J.M., Liu Y.-J., MacPherson G., Randolph G.J. (2010). Nomenclature of monocytes and dendritic cells in blood. Blood.

